# Supplementary material for: Photoinduced dynamics during electronic transfer from narrow to wide bandgap layers in one-dimensional heterostructured materials
Source: Nat Commun. 2024 May 30;15:4600. doi: 10.1038/s41467-024-48880-3 (PMC11139937; doi:10.1038/s41467-024-48880-3)
Supplement: Supplementary file 1 — Supplementary information [file 41467_2024_48880_MOESM1_ESM.pdf]

Supplementary information

"Photoinduced dynamics during electronic transfer from narrow to wide bandgap layers in one-dimensional heterostructured materials"

Y. Saida et al.

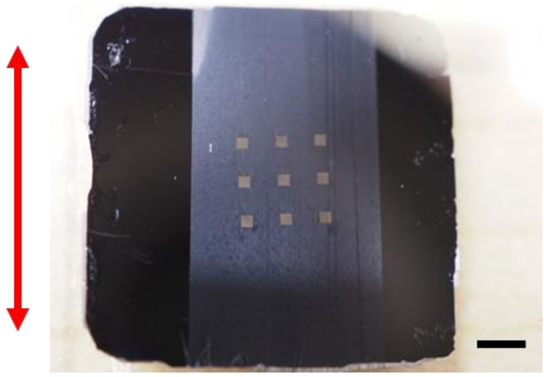

Supplementary Figure 1. Photograph of the sample used for transient absorption measurements and ultrafast electron diffraction. Carbon nanotubes (CNTs) or boron-nitride nanotubes (BNNTs) on CNTs were mounted on a sample holder made of Si substrate. Nine holes (500 × 500 μm) on the sample holder enable transmission optical spectroscopy and diffraction measurements. CNTs and BNNTs are aligned parallel to the red arrow. The black scale bar represents 2 mm.

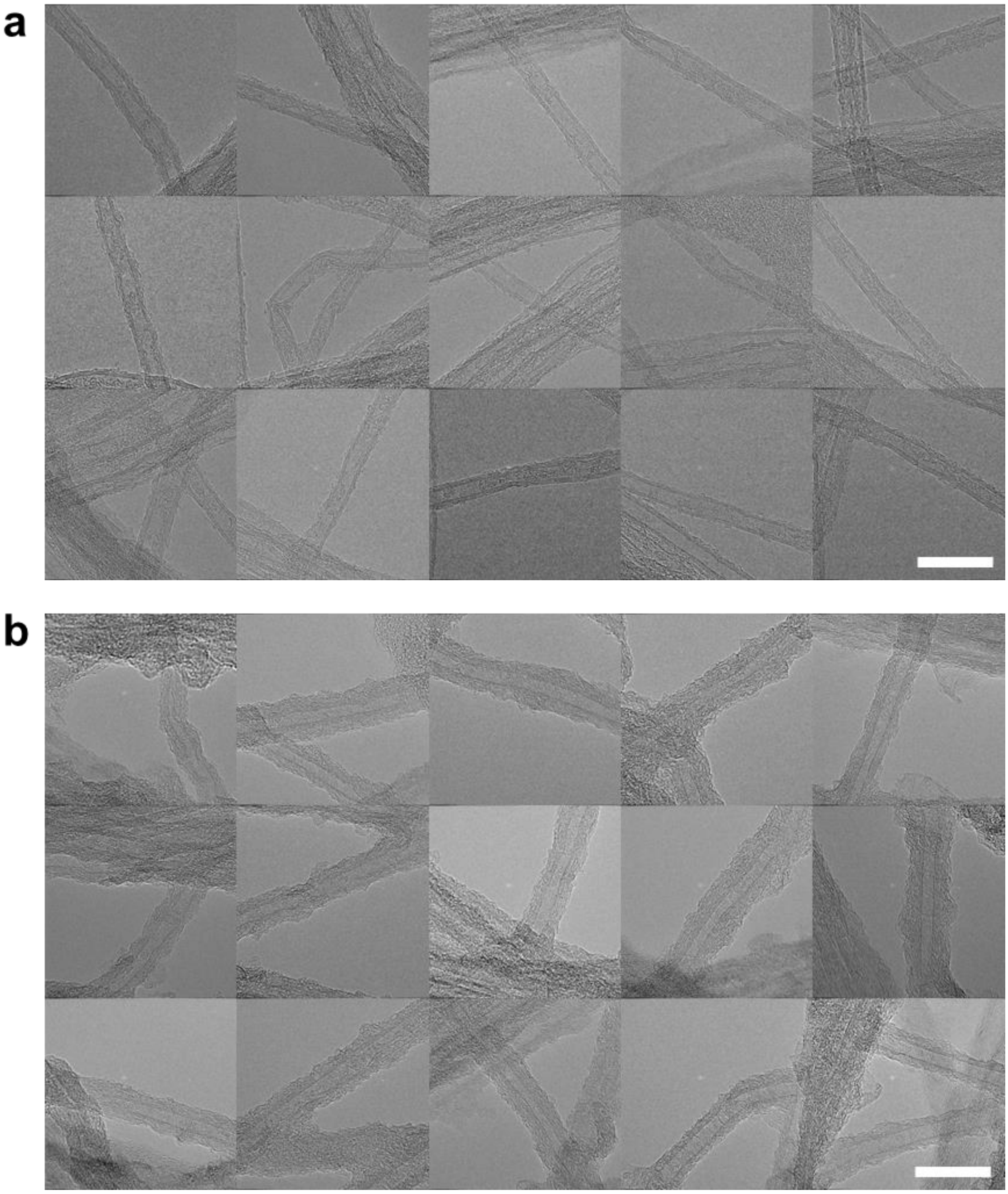

Continue to the next page.

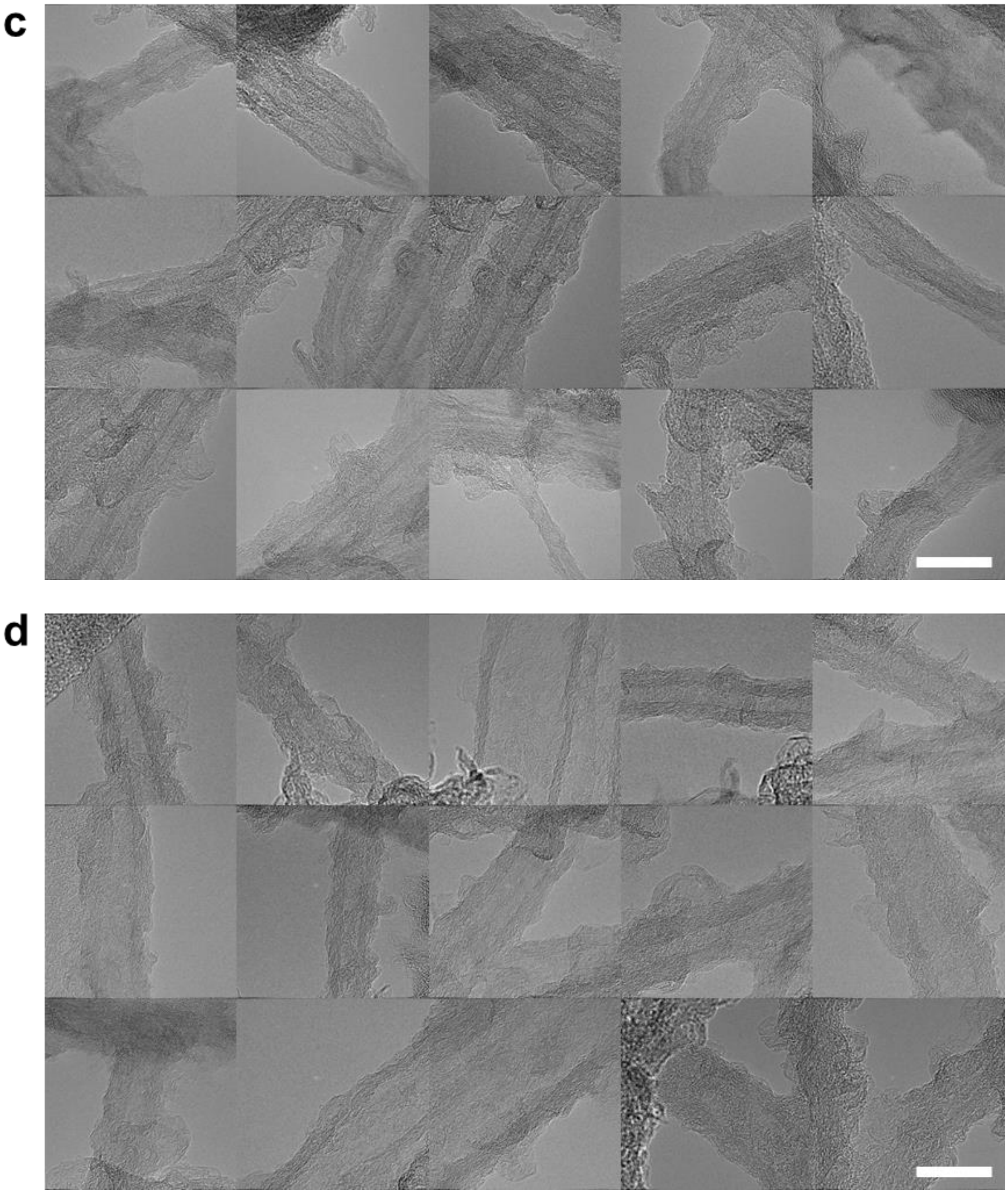

Supplementary Figure 2. Representative transmission electron microscopy (TEM) images from few-walled CNTs (**a**), few-walled CNTs with few-walled BNNTs (**b**), few-walled CNTs with multi-walled BNNTs (**c**), and few-walled BNNTs (**d**). The white scale bar represents 20 nm. TEM measurements were performed using JEM-2100F (JEOL).

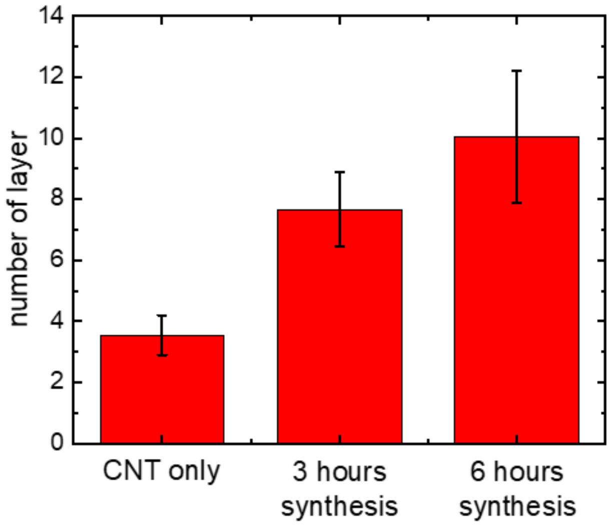

Supplementary Figure 3. Average numbers of layers of CNT (CNT without BNNT), CNT-BNNT heterostructure synthesized BNNT for 3 hours (3 hours synthesis), and 6 hours (6 hours synthesis). The number of layers was counted manually from TEM images (**Supplementary Figs. 2a–c**). Based on the data, CNTs without BNNTs have approximately 3 layers, CNT-BNNT heterostructures with synthesis for 3 hours have 3–4-layer BNNTs on the few-walled CNTs, and those with synthesis for 6 hours have ~7-layer BNNTs on the few-walled CNTs. Sample I, II, and III correspond to CNT only, 3 hours synthesis, and 6 hours synthesis, respectively. The error bars represent the standard deviation of the counted layer numbers. The numbers of CNTs or BNNTs in the samples CNT only, 3 hours synthesis, and 6 hours synthesis are n = 30, n = 36, and n = 17, respectively.

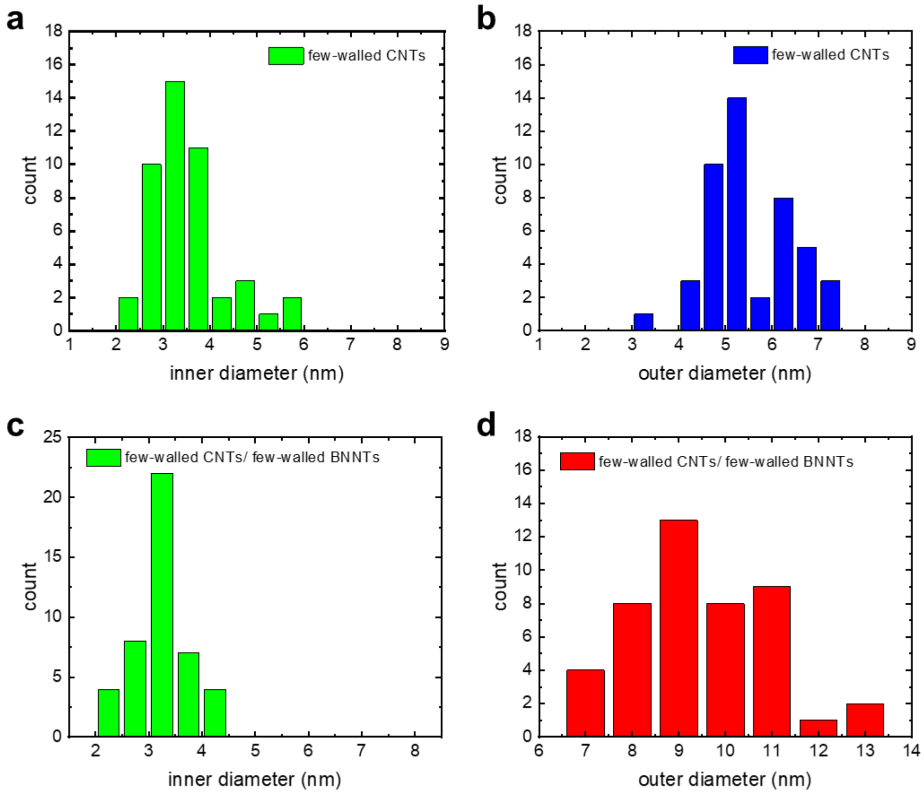

Supplementary Figure 4. Histograms of **a** inner and **b** outer diameter of CNTs obtained from **Supplementary Fig. 2a**. The average inner and outer diameter of CNTs are 3 and 5 nm, respectively. Histograms of **c** inner and **d** outer diameter of one-dimensional heterostructures of few-walled CNTs and few-walled BNNTs obtained from **Supplementary Fig. 2b**. Since the average layer distance of CNTs or BNNTs are approximately 0.34 nm, the diameter at the interface of the CNTs is approximately 5 nm, and that of counter BNNTs (diameter of the innermost layer) is estimated to be approximately 6 nm.

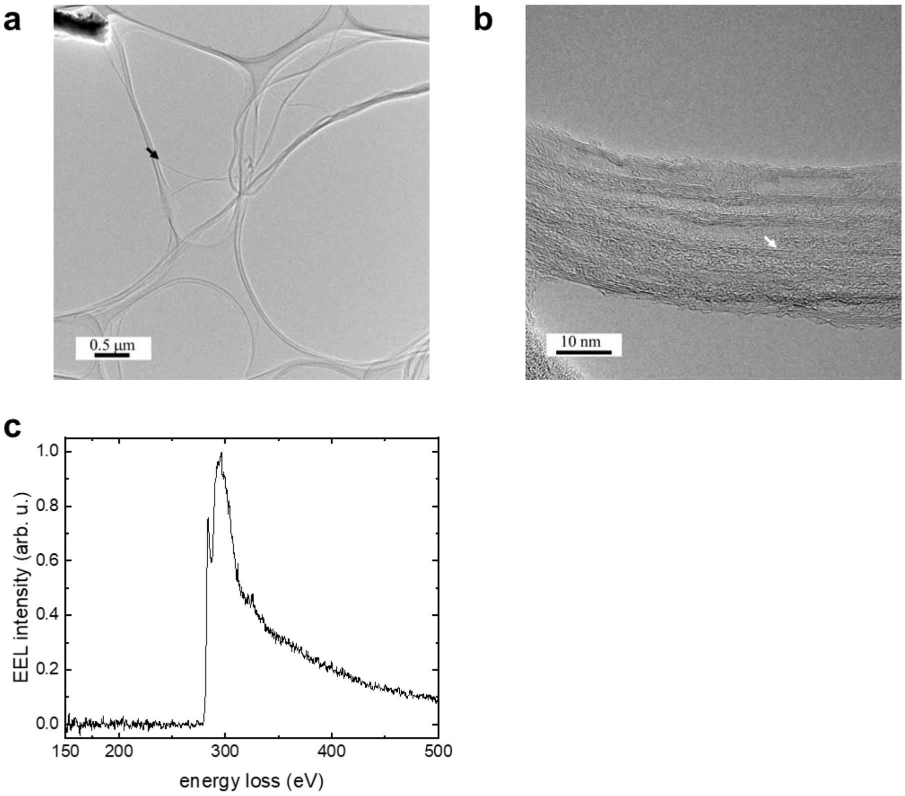

Supplementary Figure 5. TEM images (**a** and **b**) of a few-walled CNT sample (sample I) and its electron energy loss spectrum (**c**). A peak signal from carbon atom was observed. TEM electron energy loss (EEL) spectroscopy was performed using ARM-200F (cold field-emission electron gun type, JEOL). TEM image (**b)** is the enlarged view of the black arrow indicated in TEM image (**a**). The EEL spectrum (**c**) is obtained at the position indicated by the white arrow indicated in TEM image (**b**).

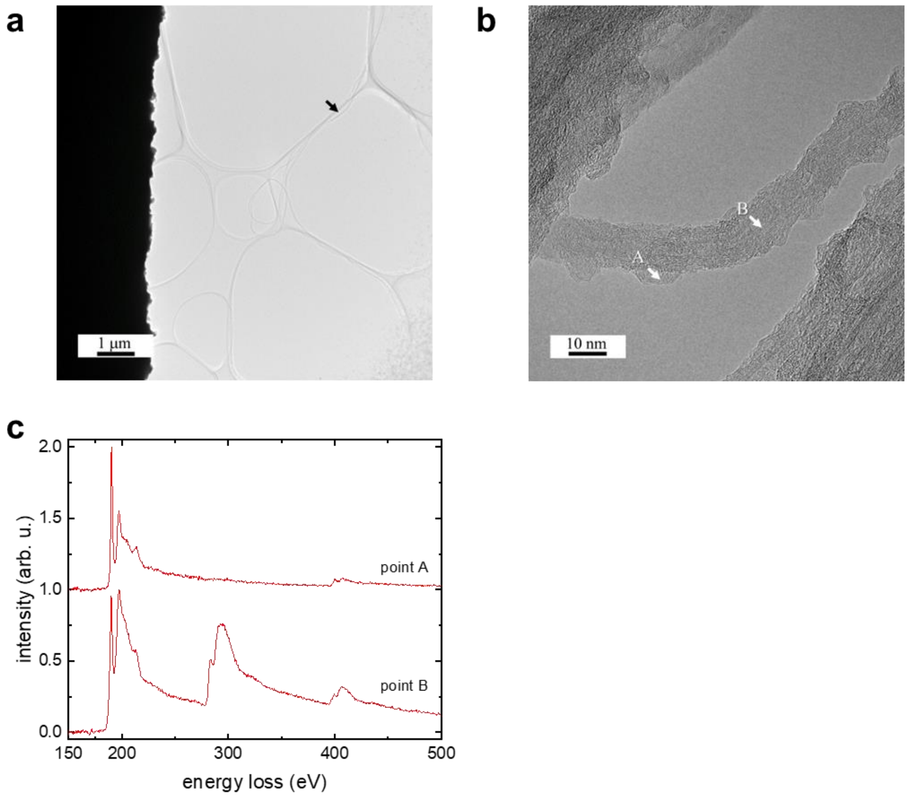

Supplementary Figure 6. TEM images (**a** and **b**) of a few-walled CNT covered with a few-walled BNNT sample (sample II) and its EEL spectra (**c**). Peak signals from boron and nitrogen atoms were observed at the outer part (A) position, and peak signals from boron, carbon, and nitrogen atoms were observed at the inner part (B) position. TEM image **(b)** is the enlarged view of the black arrow indicated in TEM image (**a**). The EEL spectra (**c**) are obtained at the position indicated by the white arrows indicated in TEM image (**b**).

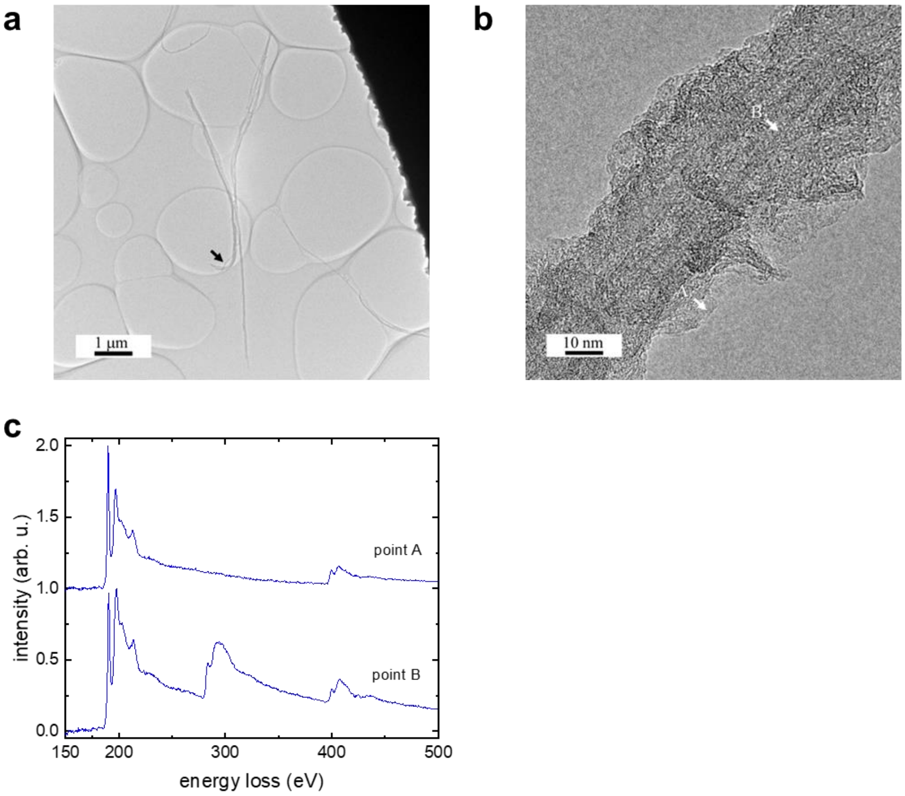

Supplementary Figure 7. TEM images (**a** and **b**) of a few-walled CNT covered with a multi-walled BNNT sample (sample III) and its EEL spectra (**c**). Peak signals from boron and nitrogen atoms were observed at the outer part (A) position, and peak signals from boron, carbon, and nitrogen atoms were observed at the inner part (B) position. TEM image (**b)** is the enlarged view of the black arrow indicated in TEM image (**a**). The EEL spectra (**c**) are obtained at the position indicated by the white arrows indicated in TEM image (**b**).

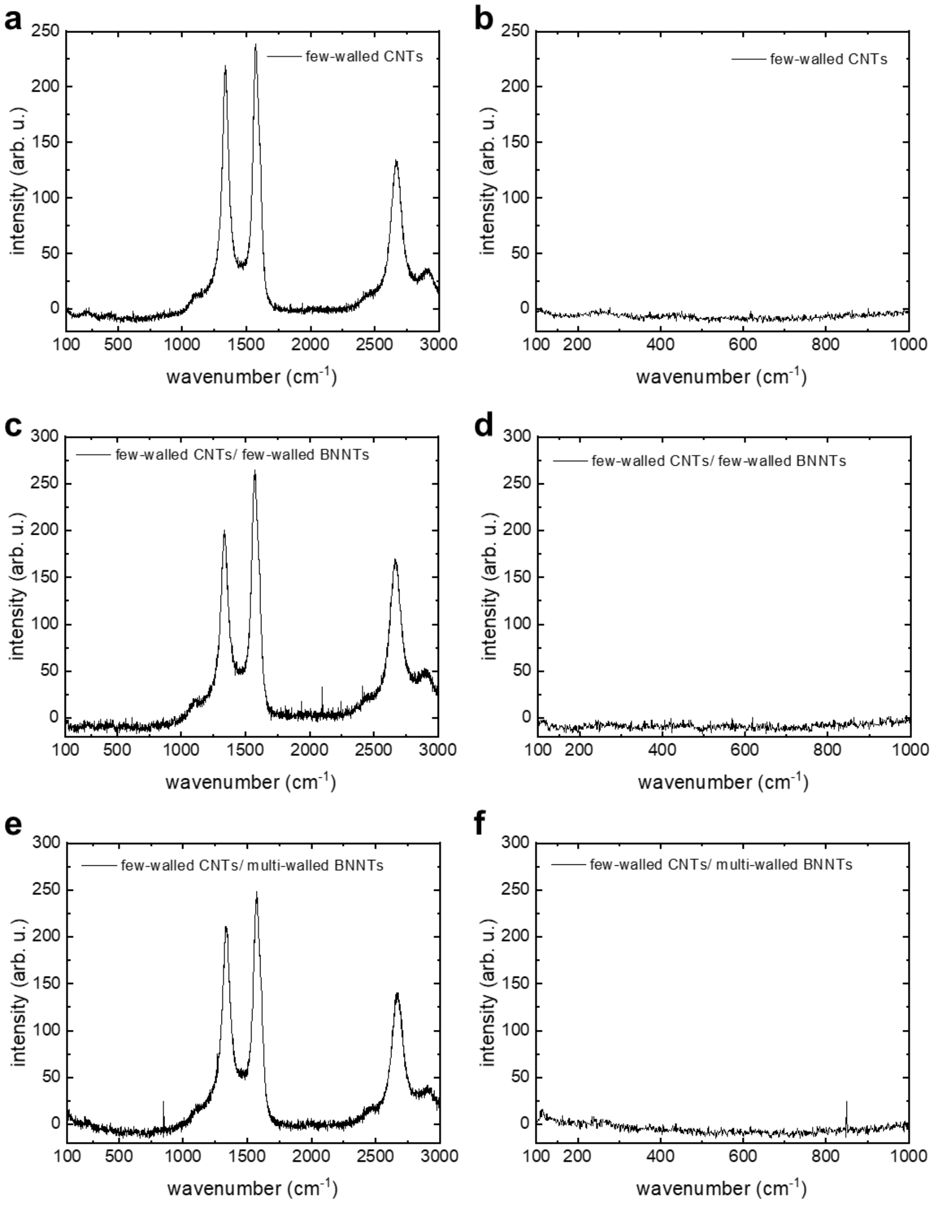

Supplementary Figure 8. Raman spectra of samples I (**a** and **b**), sample II (**c** and **d**), and sample III (**e** and **f**). Peaks from *D* (~1360 cm^{-1}), *G* (~1580 cm^{-1}), 2*D* (2700 cm^{-1}), and *D*+*G* (2900 cm^{-1}) bands were observed from the samples. However, clear peaks from radial breathing modes were not observed. Raman spectra were obtained using NRS-4500NMDS (JASCO).

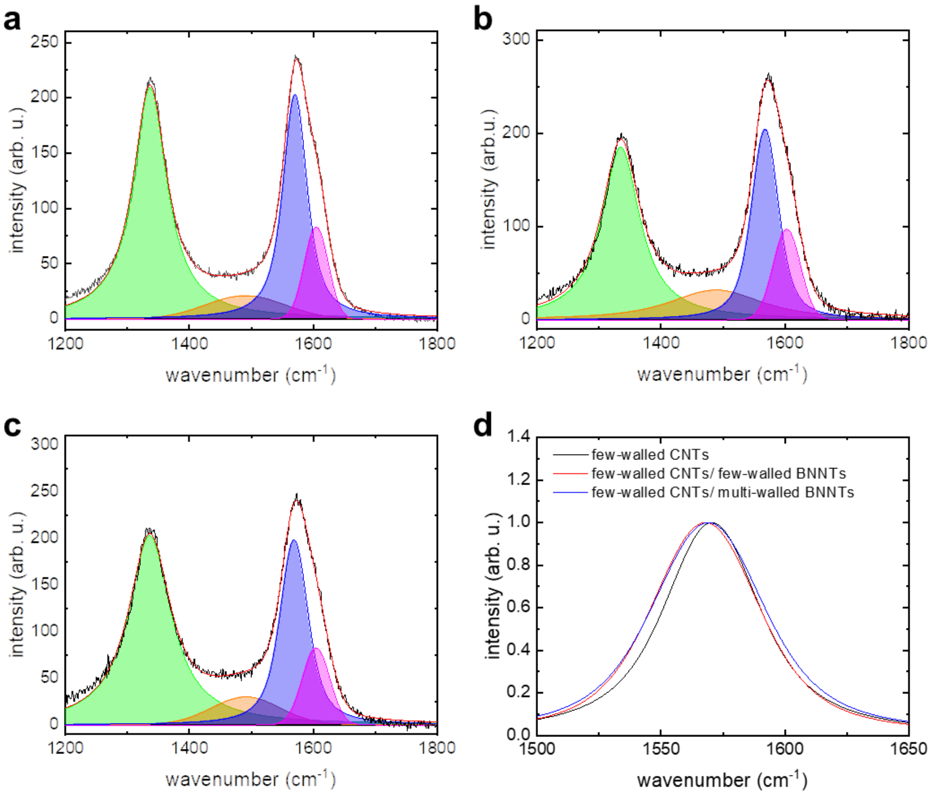

Supplementary Figure 9. Peak analyses of Raman spectra of sample I (**a**), sample II (**b**), and sample III (**c**). The black and red lines are the experimental data and fitting curves. The peaks filled in green, orange, blue, and purple are assigned to *D*-band, *D*''-band, *G*-band, and *D*'-band, respectively.[1] **d**, The peaks from the *G*-band of samples I–III. The peaks shift toward lower wavenumber for the samples with BNNTs, which is consistent with the reports of the heterostructures between carbon nanotubes and boron nitride nanotubes.[2,3]

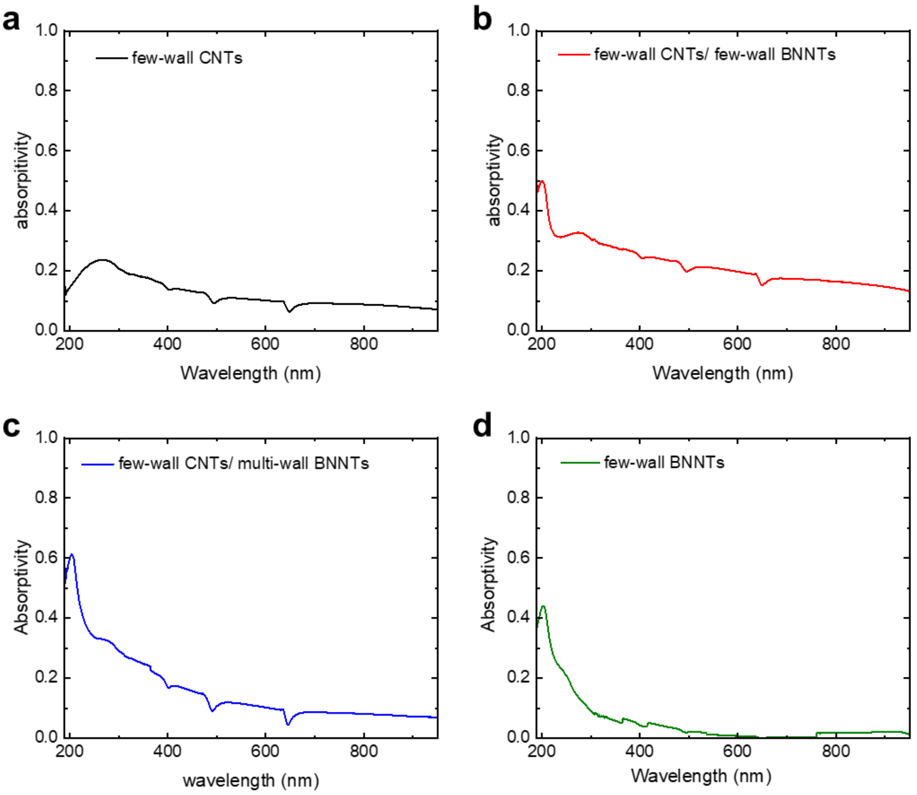

Supplementary Figure 10. Optical absorption spectra of few-walled CNTs (**a**), few-walled CNTs covered with few- (**b**) and multi-walled (**c**) BNNTs, and few-walled BNNTs (**d**) obtained by UV-vis spectrophotometer (UV1800, SHIMADZU CORPORATION). The probe light is unpolarized. CNT absorbs light over a broad spectral range spanning. BNNT presents a strong absorption band around 205 nm. The constant background from the sample frame is subtracted from the signal.

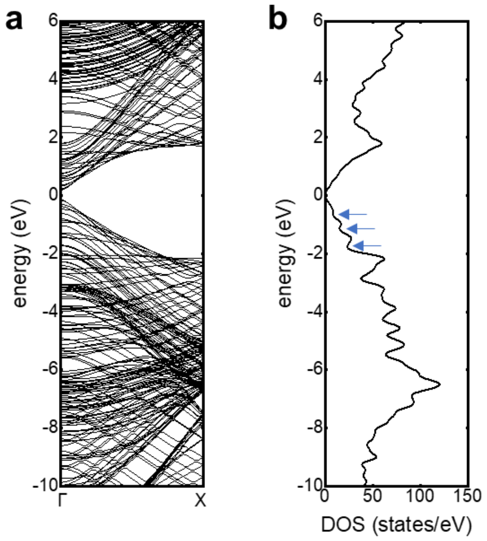

Supplementary Figure 11. a, Electronic structure of multi-walled CNTs. The bandgaps of (10,0)@(19,0)@(28,0)CNT is 0.33 eV. The energy is measured from the middle valence band maximum and the conduction band minimum. **b,** Electron DOS exhibits strong peaks around -2 and 2 eV. A few shoulders appear between these peaks, as marked with the blue arrows. The negative peaks observed in the optical absorption spectra in Supplementary Fig. 10 may reflect these shoulder structures. The atomic coordinate is shown in Supplementary Table 1.

Supplementary Table 1. Atomic coordinates (in units of Å) used for the electronic structure calculations of (10,0)@(19,0)@(28,0)CNT. The number of atoms is 228 and the lattice constant along the axis is c = 4.269135341 Å.

atom	X	Y	Z
C	3.9543094087	0.0000000000	0.0092588057
C	3.7610911919	1.2220685451	3.5650085815
C	3.7610401059	1.2220518655	2.1436591807
C	3.1991716976	2.3243468157	1.4305344028
C	3.1992162461	2.3243794790	0.0092552536
C	2.3244569173	3.1993758647	3.5650005801
C	2.3244246372	3.1993312346	2.1436651041
C	1.2219847687	3.7609047452	1.4305448349
C	1.2220014646	3.7609576691	0.0092442244
C	-0.0000305235	3.9544748287	3.5649900397
C	-0.0000307605	3.9544179855	2.1436773720
C	-1.2220379680	3.7609782898	1.4305571542
C	-1.2220554926	3.7610310181	0.0092307049
C	-2.3244213984	3.1992388631	3.5649752180
C	-2.3243897759	3.1991941899	2.1436881147
C	-3.1993795925	2.3244736501	1.4305660879
C	-3.1994247769	2.3245055829	0.0092194272
C	-3.7609068442	1.2219867269	3.5649664924
C	-3.7608554239	1.2219695237	2.1436959254
C	-3.9545643386	0.0000000000	1.4305724728
C	-3.9546202774	0.0000000000	0.0092144652
C	-3.7609068442	-1.2219867269	3.5649664924
C	-3.7608554239	-1.2219695237	2.1436959254
C	-3.1993795925	-2.3244736501	1.4305660879
C	-3.1994247769	-2.3245055829	0.0092194272
C	-2.3244213984	-3.1992388631	3.5649752180
C	-2.3243897759	-3.1991941899	2.1436881147
C	-1.2220379680	-3.7609782898	1.4305571542
C	-1.2220554926	-3.7610310181	0.0092307049
C	-0.0000305235	-3.9544748287	3.5649900397
C	-0.0000307605	-3.9544179855	2.1436773720
C	1.2219847687	-3.7609047452	1.4305448349

C	1.2220014646	-3.7609576691	0.0092442244
C	2.3244569173	-3.1993758647	3.5650005801
C	2.3244246372	-3.1993312346	2.1436651041
C	3.1991716976	-2.3243468157	1.4305344028
C	3.1992162461	-2.3243794790	0.0092552536
C	3.7610911919	-1.2220685451	3.5650085815
C	3.7610401059	-1.2220518655	2.1436591807
C	3.9542543817	0.0000000000	1.4305338948
C	7.4658609826	0.0000000000	0.0088128484
C	7.3625304019	1.2284265308	3.5654554782
C	7.3624580726	1.2284144824	2.1431278853
C	7.0573959756	2.4228073441	1.4313007034
C	7.0574659683	2.4228310818	0.0084532105
C	6.5638533272	3.5524085047	3.5655191376
C	6.5637862955	3.5523695661	2.1430664317
C	5.8912837834	4.5855252020	1.4309446339
C	5.8913460352	4.5855730346	0.0088135053
C	5.0558440811	5.4919795623	3.5654088731
C	5.0557899304	5.4919209649	2.1431797779
C	4.0813495729	6.2469754316	1.4312679852
C	4.0813927504	6.2470402063	0.0084883079
C	2.9976606552	6.8344929296	3.5655891749
C	2.9976303731	6.8344213146	2.1429948125
C	1.8325252780	7.2371623817	1.4309518629
C	1.8325446009	7.2372409914	0.0088006117
C	0.6166183845	7.4400205976	3.5653750771
C	0.6166104673	7.4399423106	2.1432088430
C	-0.6161359504	7.4372229599	1.4312076681
C	-0.6161405912	7.4373016615	0.0085449107
C	-1.8319598490	7.2340335992	3.5656522432
C	-1.8319400558	7.2339609373	2.1429285288
C	-2.9987573355	6.8362215100	1.4309759841
C	-2.9987875859	6.8362912246	0.0087756760
C	-4.0832040327	6.2500541838	3.5653597665
C	-4.0831621796	6.2499902644	2.1432241849
C	-5.0545365871	5.4909828616	1.4311392464

C	-5.0545881080	5.4910383887	0.0086143573
C	-5.8884890427	4.5831905824	3.5656998243
C	-5.8884284312	4.5831424215	2.1428812789
C	-6.5648362394	3.5525549752	1.4310131484
C	-6.5649051870	3.5525904016	0.0087352576
C	-7.0611875513	2.4241649944	3.5653509186
C	-7.0611103358	2.4241388600	2.1432263608
C	-7.3620590763	1.2286914078	1.4310676443
C	-7.3621406749	1.2287056561	0.0086761784
C	-7.4618583691	0.0000000000	3.5657136147
C	-7.4617749185	0.0000000000	2.1428588609
C	-7.3620590763	-1.2286914078	1.4310676443
C	-7.3621406749	-1.2287056561	0.0086761784
C	-7.0611875513	-2.4241649944	3.5653509186
C	-7.0611103358	-2.4241388600	2.1432263608
C	-6.5648362394	-3.5525549752	1.4310131484
C	-6.5649051870	-3.5525904016	0.0087352576
C	-5.8884890427	-4.5831905824	3.5656998243
C	-5.8884284312	-4.5831424215	2.1428812789
C	-5.0545365871	-5.4909828616	1.4311392464
C	-5.0545881080	-5.4910383887	0.0086143573
C	-4.0832040327	-6.2500541838	3.5653597665
C	-4.0831621796	-6.2499902644	2.1432241849
C	-2.9987573355	-6.8362215100	1.4309759841
C	-2.9987875859	-6.8362912246	0.0087756760
C	-1.8319598490	-7.2340335992	3.5656522432
C	-1.8319400558	-7.2339609373	2.1429285288
C	-0.6161359504	-7.4372229599	1.4312076681
C	-0.6161405912	-7.4373016615	0.0085449107
C	0.6166183845	-7.4400205976	3.5653750771
C	0.6166104673	-7.4399423106	2.1432088430
C	1.8325252780	-7.2371623817	1.4309518629
C	1.8325446009	-7.2372409914	0.0088006117
C	2.9976606552	-6.8344929296	3.5655891749
C	2.9976303731	-6.8344213146	2.1429948125
C	4.0813495729	-6.2469754316	1.4312679852

C	4.0813927504	-6.2470402063	0.0084883079
C	5.0558440811	-5.4919795623	3.5654088731
C	5.0557899304	-5.4919209649	2.1431797779
C	5.8912837834	-4.5855252020	1.4309446339
C	5.8913460352	-4.5855730346	0.0088135053
C	6.5638533272	-3.5524085047	3.5655191376
C	6.5637862955	-3.5523695661	2.1430664317
C	7.0573959756	-2.4228073441	1.4313007034
C	7.0574659683	-2.4228310818	0.0084532105
C	7.3625304019	-1.2284265308	3.5654554782
C	7.3624580726	-1.2284144824	2.1431278853
C	7.4657894864	0.0000000000	1.4309407174
C	11.0023353044	0.0000000000	0.0085175601
C	10.9294692204	1.2306440906	3.5656819043
C	10.9294139801	1.2306388594	2.1428454005
C	10.7152317526	2.4447156772	1.4314074087
C	10.7152862310	2.4447295214	0.0082982181
C	10.3695176122	3.6282137291	3.5658685626
C	10.3694696575	3.6281978370	2.1426577780
C	9.9001642653	4.7683799900	1.4314304630
C	9.9002099088	4.7683992568	0.0082763825
C	9.3104237811	5.8510499533	3.5657198966
C	9.3103820856	5.8510242355	2.1428115162
C	8.6011412565	6.8593896071	1.4311983671
C	8.6011790568	6.8594206287	0.0085138321
C	7.7780835861	7.7772037772	3.5656569562
C	7.7780463447	7.7771689154	2.1428769136
C	6.8538346257	8.5930698490	1.4313877388
C	6.8538637785	8.5931081246	0.0083210856
C	5.8450804152	9.3019825440	3.5658649198
C	5.8450558206	9.3019390218	2.1426606335
C	4.7666546756	9.8996960211	1.4314397438
C	4.7666765693	9.8997409308	0.0082590620
C	3.6304473113	10.3783194759	3.5657444816
C	3.6304311334	10.3782697989	2.1427729289
C	2.4475947766	10.7256917065	1.4312027263

C	2.4476055073	10.7257438270	0.0084954995
C	1.2321708614	10.9318276886	3.5656240637
C	1.2321633679	10.9317754227	2.1428988718
C	0.0009330977	10.9940963962	1.4313551291
C	0.0009332029	10.9941505044	0.0083492439
C	-1.2296293513	10.9181717860	3.5658555605
C	-1.2296214761	10.9181188896	2.1426705804
C	-2.4450790055	10.7110305334	1.4314514937
C	-2.4450906576	10.7110861252	0.0082536378
C	-3.6315296690	10.3757394000	3.5657800802
C	-3.6315141406	10.3756889607	2.1427443075
C	-4.7732032860	9.9105004400	1.4312295859
C	-4.7732257592	9.9105444729	0.0084737512
C	-5.8528077657	9.3153290195	3.5656080291
C	-5.8527845138	9.3152874287	2.1429175277
C	-6.8547682811	8.5970687880	1.4313242233
C	-6.8547970245	8.5971056707	0.0083798317
C	-7.7690585175	7.7699953485	3.5658449850
C	-7.7690217071	7.7699607634	2.1426792874
C	-8.5890426373	6.8492039881	1.4314564226
C	-8.5890799224	6.8492341779	0.0082446184
C	-9.3068826556	5.8467803237	3.5658039907
C	-9.3068411578	5.8467538217	2.1427172770
C	-9.9099784915	4.7715459782	1.4312546539
C	-9.9100250749	4.7715648803	0.0084433800
C	-10.3845926915	3.6337648108	3.5655952902
C	-10.3845417423	3.6337468709	2.1429225767
C	-10.7215337228	2.4479050740	1.4312858579
C	-10.7215864895	2.4479181123	0.0084117465
C	-10.9204937313	1.2312286640	3.5658243977
C	-10.9204419031	1.2312235691	2.1426959418
C	-10.9858431021	0.0000000000	1.4314568752
C	-10.9858960927	0.0000000000	0.0082432100
C	-10.9204937313	-1.2312286640	3.5658243977
C	-10.9204419031	-1.2312235691	2.1426959418
C	-10.7215337228	-2.4479050740	1.4312858579

C	-10.7215864895	-2.4479181123	0.0084117465
C	-10.3845926915	-3.6337648108	3.5655952902
C	-10.3845417423	-3.6337468709	2.1429225767
C	-9.9099784915	-4.7715459782	1.4312546539
C	-9.9100250749	-4.7715648803	0.0084433800
C	-9.3068826556	-5.8467803237	3.5658039907
C	-9.3068411578	-5.8467538217	2.1427172770
C	-8.5890426373	-6.8492039881	1.4314564226
C	-8.5890799224	-6.8492341779	0.0082446184
C	-7.7690585175	-7.7699953485	3.5658449850
C	-7.7690217071	-7.7699607634	2.1426792874
C	-6.8547682811	-8.5970687880	1.4313242233
C	-6.8547970245	-8.5971056707	0.0083798317
C	-5.8528077657	-9.3153290195	3.5656080291
C	-5.8527845138	-9.3152874287	2.1429175277
C	-4.7732032860	-9.9105004400	1.4312295859
C	-4.7732257592	-9.9105444729	0.0084737512
C	-3.6315296690	-10.3757394000	3.5657800802
C	-3.6315141406	-10.3756889607	2.1427443075
C	-2.4450790055	-10.7110305334	1.4314514937
C	-2.4450906576	-10.7110861252	0.0082536378
C	-1.2296293513	-10.9181717860	3.5658555605
C	-1.2296214761	-10.9181188896	2.1426705804
C	0.0009330977	-10.9940963962	1.4313551291
C	0.0009332029	-10.9941505044	0.0083492439
C	1.2321708614	-10.9318276886	3.5656240637
C	1.2321633679	-10.9317754227	2.1428988718
C	2.4475947766	-10.7256917065	1.4312027263
C	2.4476055073	-10.7257438270	0.0084954995
C	3.6304473113	-10.3783194759	3.5657444816
C	3.6304311334	-10.3782697989	2.1427729289
C	4.7666546756	-9.8996960211	1.4314397438
C	4.7666765693	-9.8997409308	0.0082590620
C	5.8450804152	-9.3019825440	3.5658649198
C	5.8450558206	-9.3019390218	2.1426606335
C	6.8538346257	-8.5930698490	1.4313877388

C	6.8538637785	-8.5931081246	0.0083210856
C	7.7780835861	-7.7772037772	3.5656569562
C	7.7780463447	-7.7771689154	2.1428769136
C	8.6011412565	-6.8593896071	1.4311983671
C	8.6011790568	-6.8594206287	0.0085138321
C	9.3104237811	-5.8510499533	3.5657198966
C	9.3103820856	-5.8510242355	2.1428115162
C	9.9001642653	-4.7683799900	1.4314304630
C	9.9002099088	-4.7683992568	0.0082763825
C	10.3695176122	-3.6282137291	3.5658685626
C	10.3694696575	-3.6281978370	2.1426577780
C	10.7152317526	-2.4447156772	1.4314074087
C	10.7152862310	-2.4447295214	0.0082982181
C	10.9294692204	-1.2306440906	3.5656819043
C	10.9294139801	-1.2306388594	2.1428454005
C	11.0022792944	0.0000000000	1.4311889415

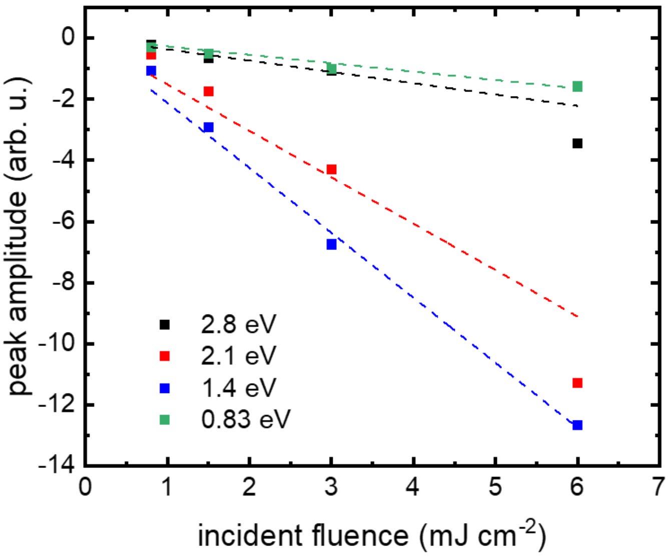

Supplementary Figure 12. The incident fluence dependence of the peak amplitude of transient absorption signals of multi-walled BNNT on few-walled CNT. The absolute value of the signal linearly increases with the incident fluence in the fluence range below 6 mJ cm^{-2}.

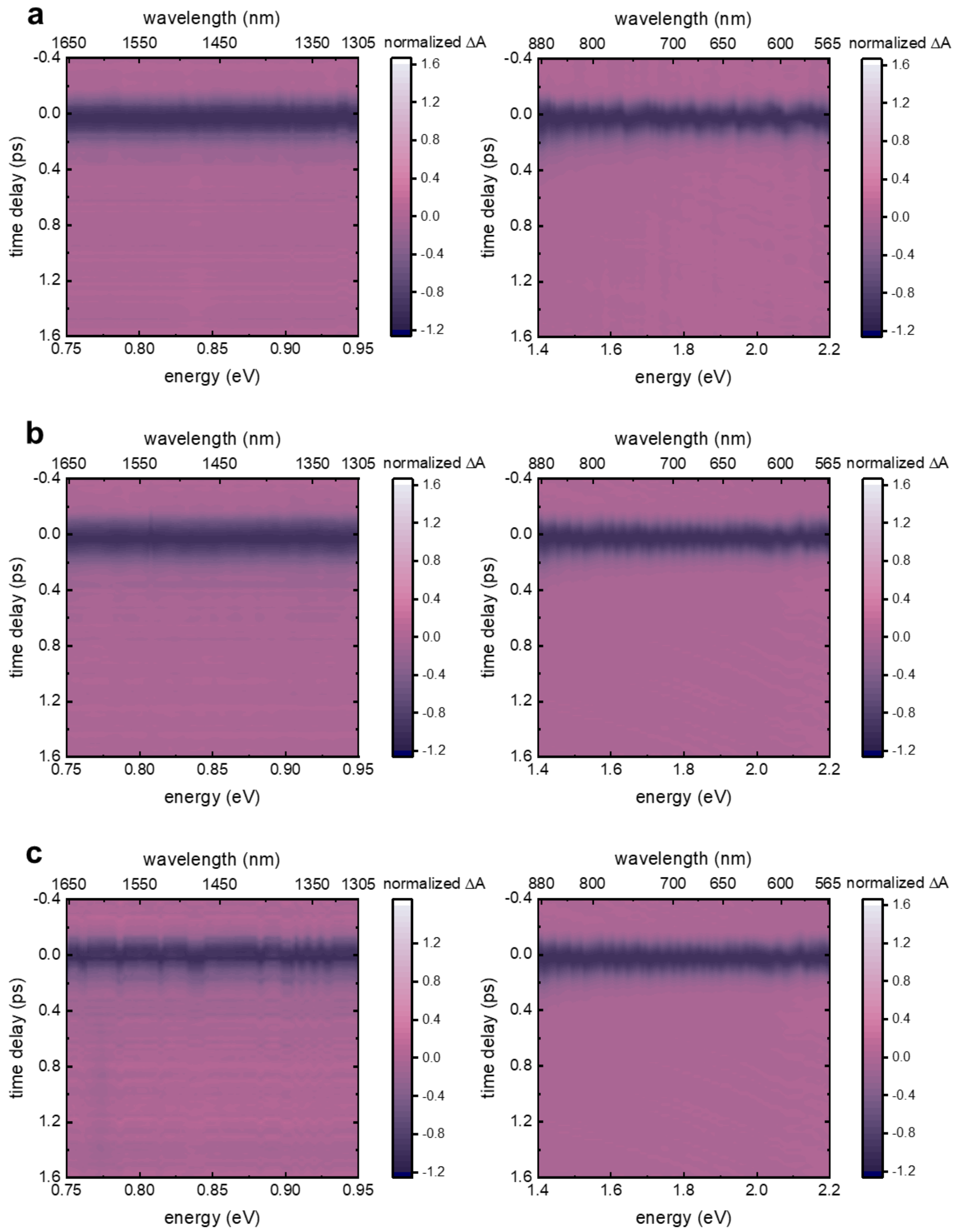

Supplementary Figure 13. Transient absorption spectra of few-walled CNT (**a**), few-walled BNNT on few-walled CNT (**b**), and multi-walled BNNT on few-walled CNT (**c**) in visible to IR spectral range following femtosecond excitation with 400 nm pump pulse. The left panels correspond to IR regions, and the right panels correspond to visible regions. The figure shows that the electronic relaxation occurs within CNTs, and the pump-probe signals in all the samples are almost identical in this spectral range.

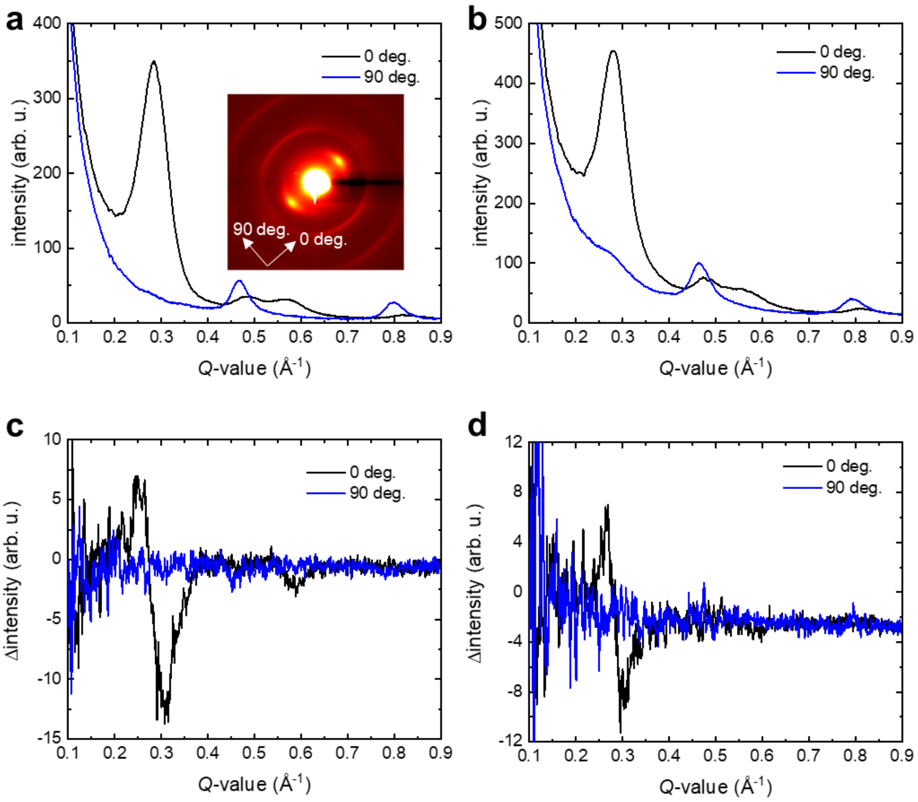

Supplementary Figure 14. Radial average of the electron diffraction pattern from samples II (**a**) and III (**b**). In the legend, 0 deg. is the direction to the broad spots as shown in the inset of the figure. The differential diffraction patterns from samples II (**c**) and III (**d**) at the time delay of +150 ps. The peak shift to the lower *Q*-value is observed in **c** and **d** at around the *Q*-value of 0.3 $Å^{-1}$.

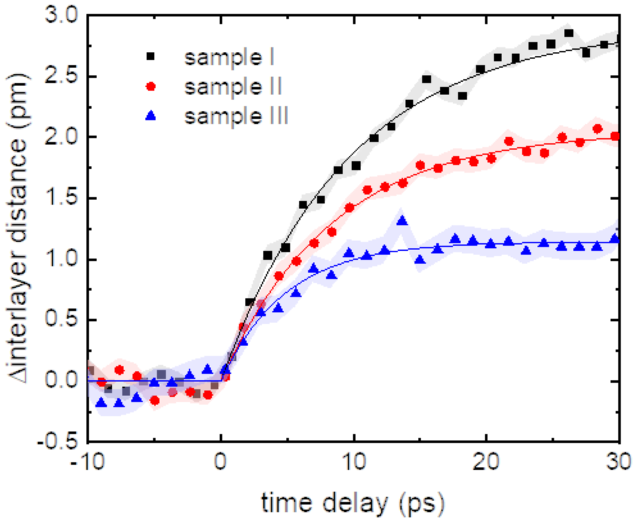

Supplementary Figure 15. Changes in the interlayer distance of samples I, II, and III. The absorbed fluence of the samples is similar since BNNTs do not absorb near-UV light. The number of layers of samples increases in the order of I, II, and III, as shown in Supplementary Fig. 3. The amplitude of the interlayer distance decreases in the order of samples I, II, and III because the absorbed fluence is distributed to more layers for sample III than samples I and II. The error bars indicated by the shaded area represent the standard deviation at each time delay points (n = 12).

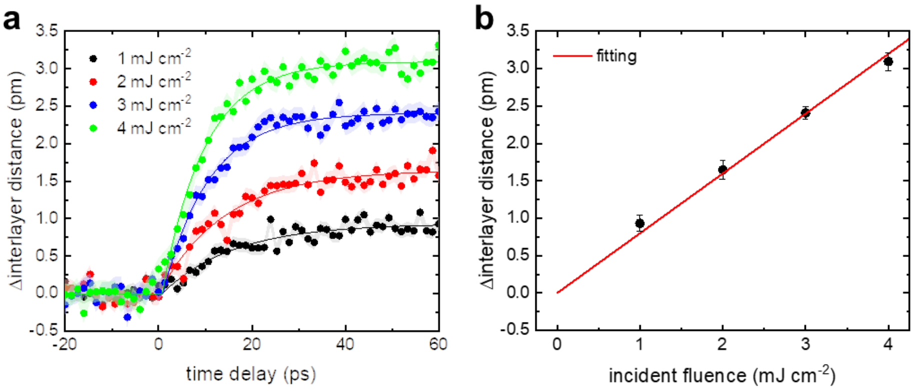

Supplementary Figure 16. a, Time evolution of interlayer distance depending on the pump pulse fluence observed for sample II. The interlayer distance is obtained from the Gaussian fit of the radial average of diffraction patterns. Solid lines are fitting curves with Eq. 2. The error bars indicated by the shaded area represent the standard deviation at each time delay points (n = 25). **b,** Intensity dependence of the maximum changes in interlayer distance. The changes in interlayer distance are derived from a, at the data point from 40 ps to 66.7 ps. The interlayer expansion is linear with the incident fluence, suggesting that the two-photon absorption process does not occur within this range of excitation density. The error bars represent the standard deviation at the data points (n = 21).

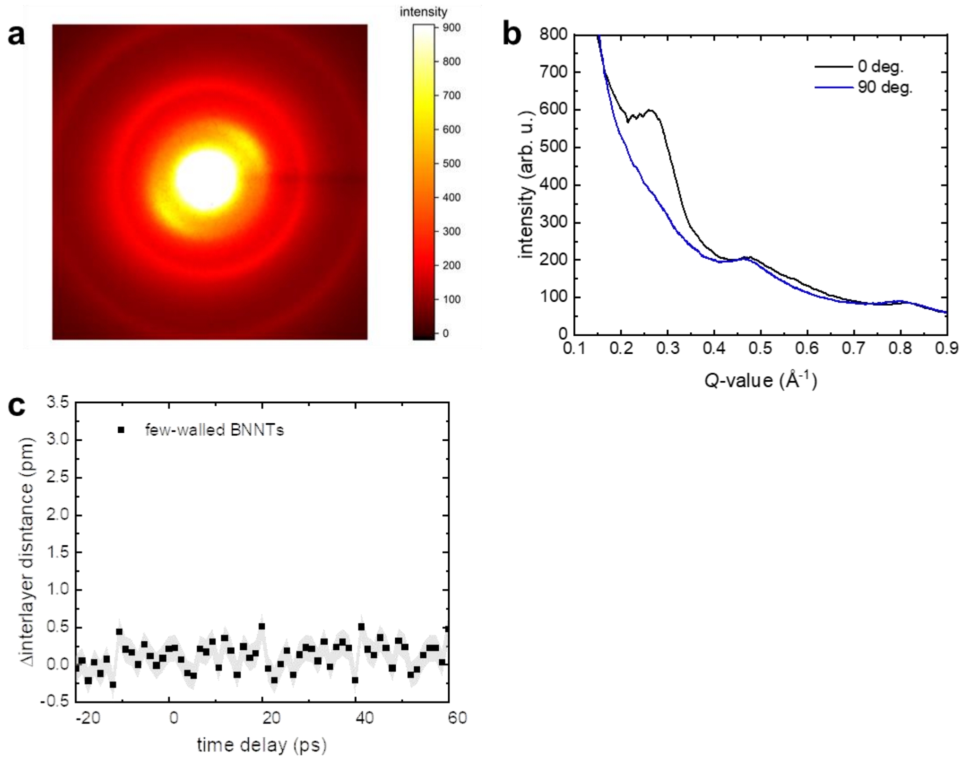

Supplementary Figure 17. a, Electron diffraction pattern from few-walled BNNTs. **b,** a radial average of the diffraction pattern. 0 deg. is the direction to the broad spots, the same as supplementary Fig. 11a. **c**, Time evolution of interlayer distance observed in **a**. The error bars indicated by the shaded area represent the standard deviation at each time delay points (n = 30).

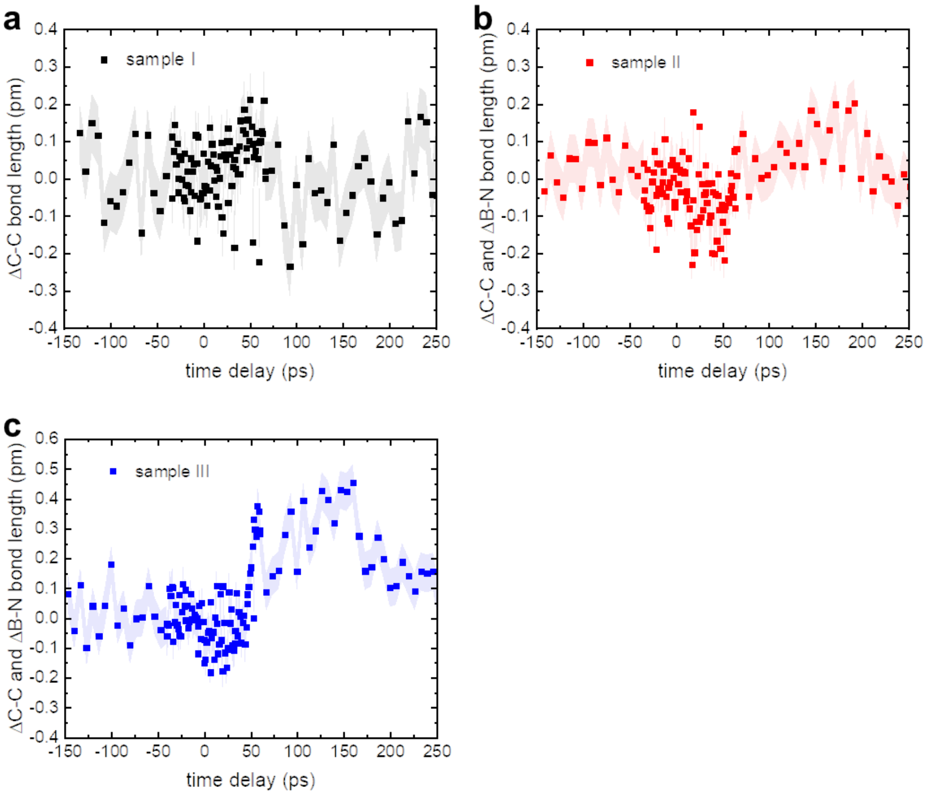

Supplementary Figure 18. Time evolution of C–C and B–N bond lengths after photoexcitation of sample I (**a**), II (**b**), and III (**c**). The bond lengths are obtained from the Gaussian fit of the (100) and (110) plane distance in the radial average of the electron diffraction pattern. The average C–C bond length of the few-walled CNTs remains constant after 400 nm photoexcitation. However, the C–C and B–N bond lengths of the CNT-BNNT heterostructure are modified following femtosecond photoexcitation. It shows an average decrease in <10 ps and a gradual increase in ~100 ps. Bond shrinks in CNT-BNNT heterostructure suggest that negatively charged boron and nitrogen atoms attracted each other in <10 ps and relaxed in ~100 ps. The error bars indicated by the shaded area represent the standard deviation at each time delay points (n = 45 for a, n = 48 for b, and n = 52 for c).

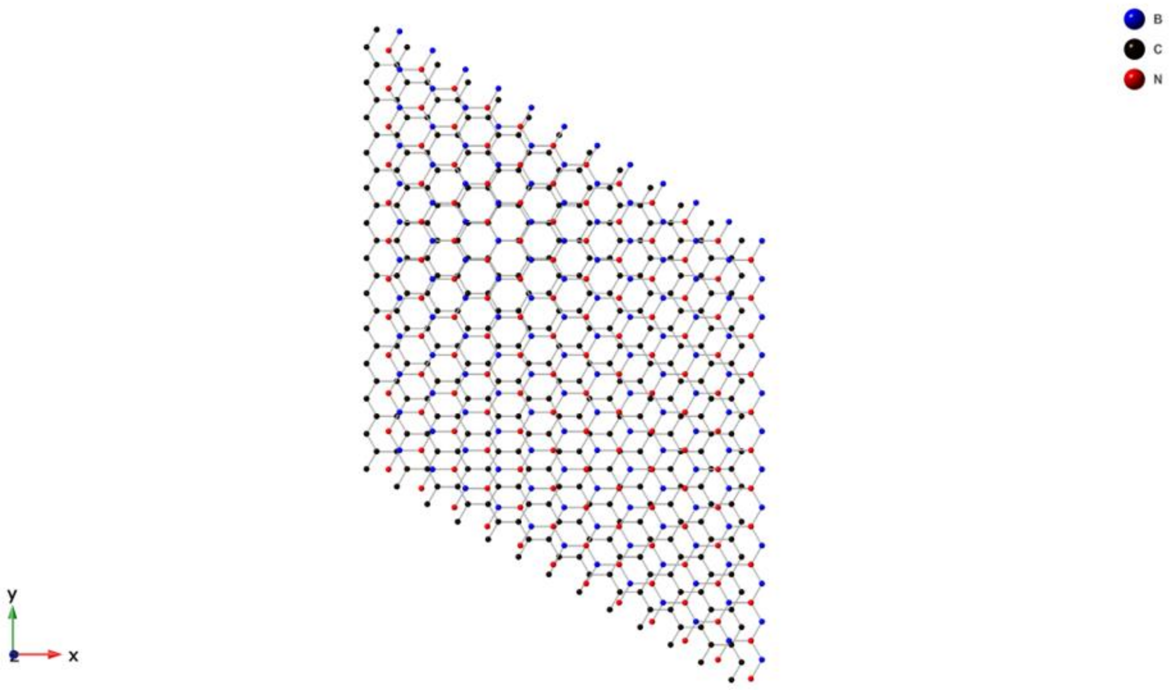

Supplementary Fig. 19. Model structure of h-BN on graphene. Under the periodical boundary condition, 12×12 unit-cells of h-BN are placed on 13 × 13 unit-cells of graphene to induce a lattice mismatch between the layers. The initial layer distance is set to 3.6–3.7 Å. Before the TDDFT calculation explained in Supplementary Note 2, the model structure is optimized at room temperature.

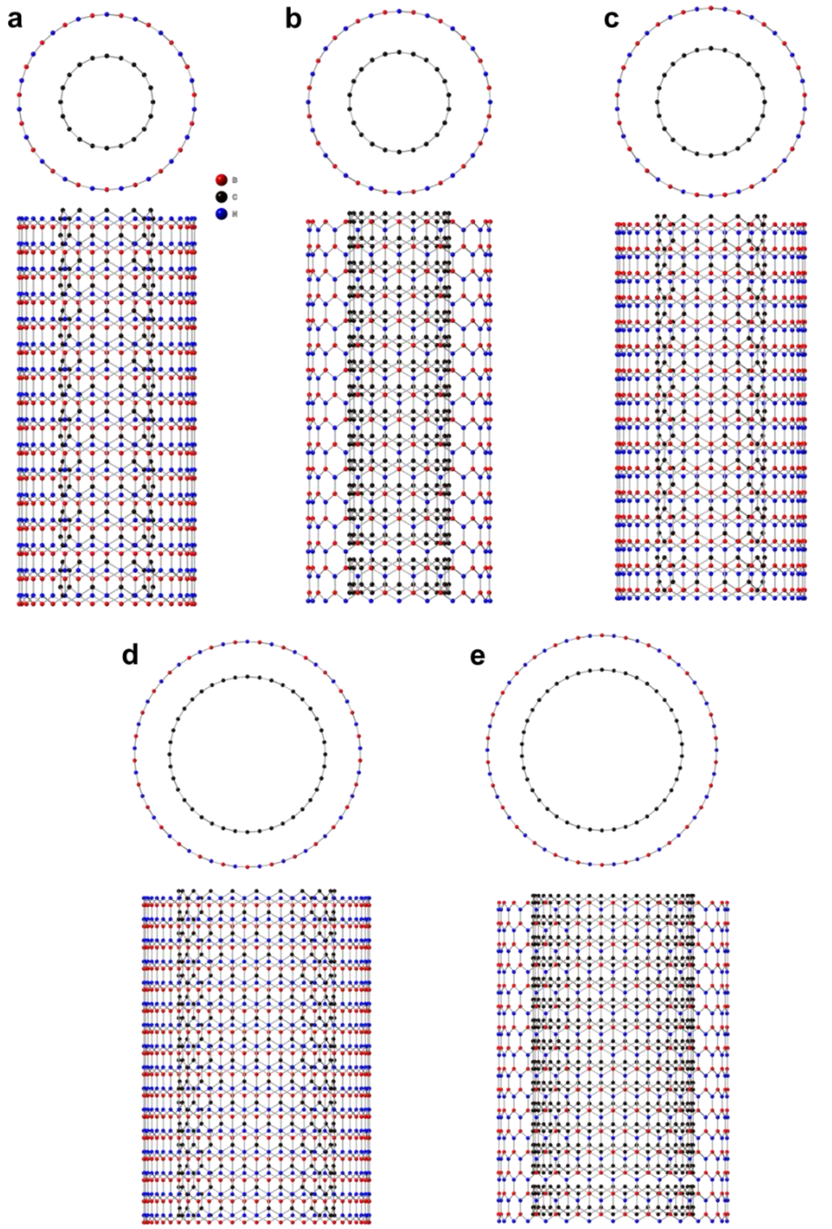

Supplementary Fig. 20. Model structures of single-walled CNT covered by single-walled BNNT. The 1D periodical boundary condition to the CNT long axis is applied for these model structures. The diameters of inner CNT are approximately 1 nm for **a** CNT(10,0)@BNNT(19,0), **b** CNT(11,0)@BNNT(20,0), and **c** CNT(12,0)@BNNT(21,0) and those are approximately 1.6 nm for **d** CNT(20,0)@BNNT(29,0), and **e** CNT(21,0)@BNNT(30,0). The upper and lower panels show the top and side views of CNT/BNNTs, respectively. The initial layer distance is approximately 3.5 Å. Before the TDDFT calculation explained in Supplementary Note 2, the model structure is optimized at room temperature.

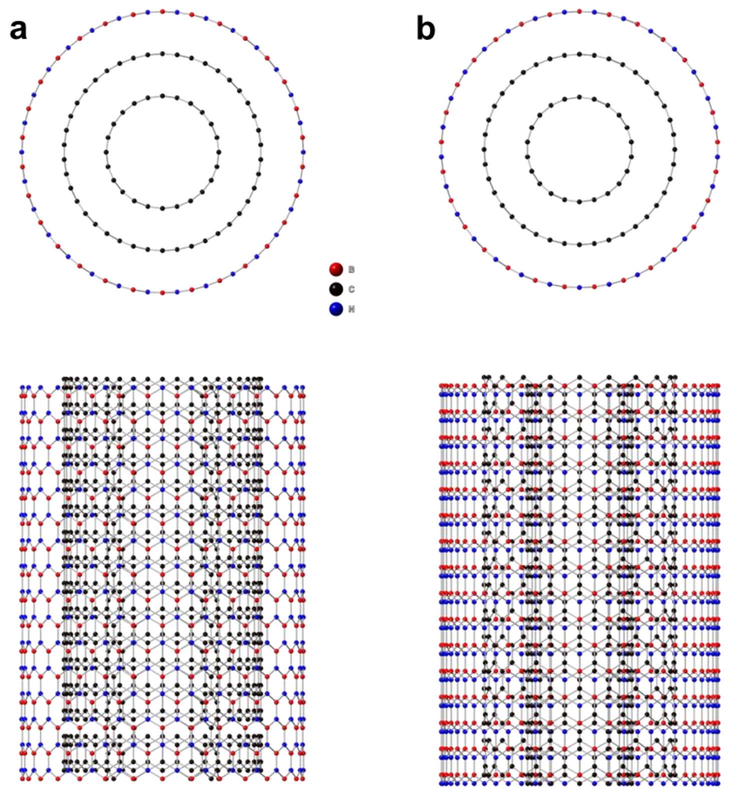

Supplementary Fig. 21. Model structures of double-walled CNT covered by single-walled BNNT. The 1D periodical boundary condition to the CNT long axis is applied for these model structures. The diameters of CNTs are approximately 1 and 1.6 nm for **a** CNT(11,0)@CNT(20,0)@BNNT(29,0) and **b** CNT(12,0)@CNT(21,0)@BNNT(30,0). The upper and lower panels show the top and side views of CNT/BNNTs, respectively. The initial layer distance is approximately 3.5 Å. Before the TDDFT calculation explained in Supplementary Note 2, the model structure is optimized at room temperature.

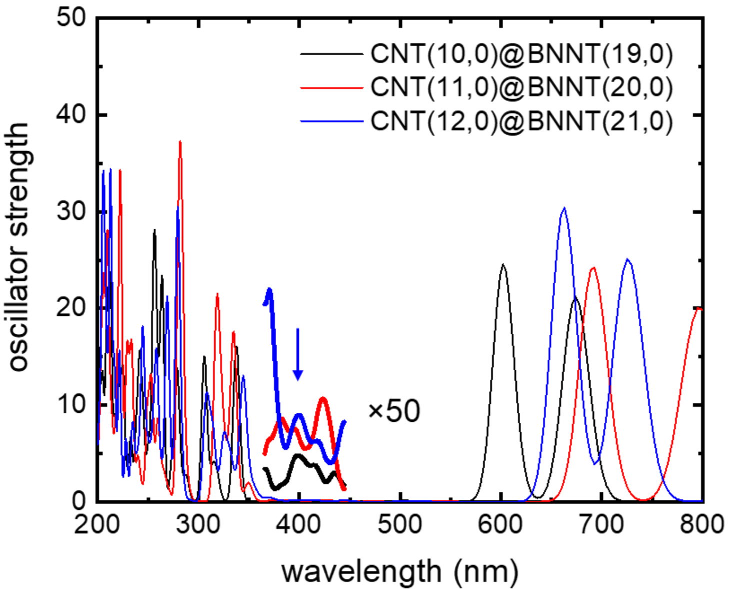

Supplementary Fig. 22. Oscillator strength for the electronic transition for CNT/BNNT heterostructures with the interfacial CNT diameter of approximately 1.0 nm (CNT(10,0)@BNNT(19,0), CNT(11,0)@BNNT(20,0), and CNT(12,0)@BNNT(21,0)). The electron transition at the wavelength of 400 nm (marked with the blue arrow) is significantly weak compared with larger diameter CNT/BNNT structures.

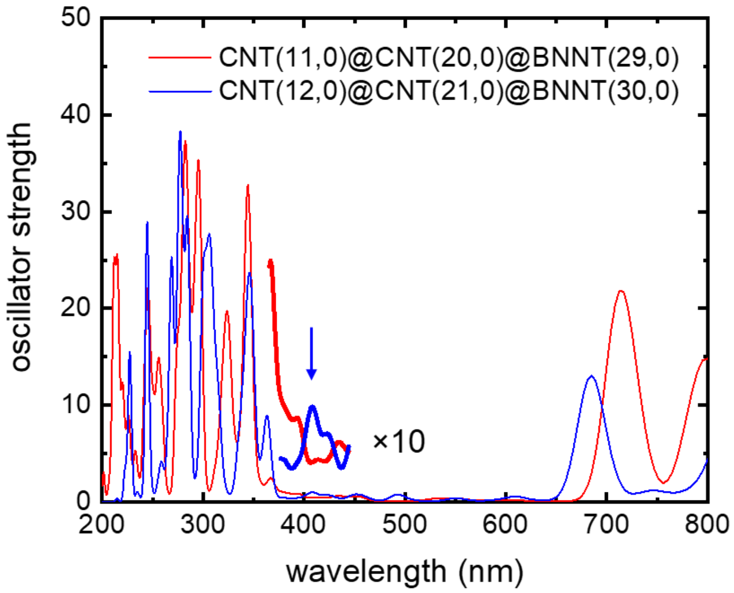

Supplementary Fig. 23. Oscillator strength for the electronic transition for a double-walled CNT and a single-walled BNNT heterostructures (CNT(11,0)@CNT(20,0)@BNNT(29,0) and CNT(12,0)@CNT(21,0)@BNNT(30,0)). The electron transition of a double-walled CNT and a single-walled BNNT heterostructures at a wavelength of 400 nm (marked with the blue arrow) is observed and is similar amplitude to that of a single-walled CNT and a single-walled BNNT heterostructures (CNT diameter of 1.6 nm). The effect regarding the layer stacking of CNTs is much smaller than that of the interlayer diameter (curvature) of CNTs, which is consistent with a previous report.[4]

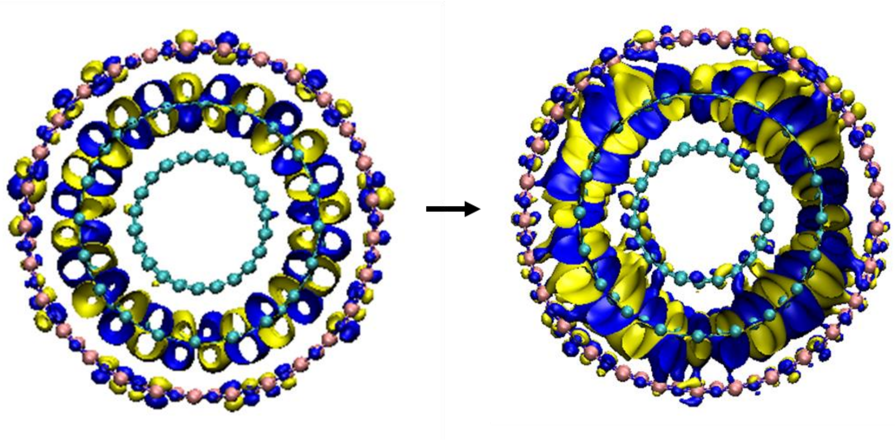

Supplementary Fig. 24. Electronic wavefunction of a double-walled CNT covered with single-walled BNNT heterostructure at an energy of 3.1 eV before and after photoexcitation. Overlapping of electronic wavefunction between CNT layers and BNNT layer is not observed before photoexcitation but is observed after photoexcitation. The wavefunctions represent the Kohn-Sham orbitals at the unoccupied states corresponding to the excited states in the transition at the peaks of the oscillator strength, where the yellow and blue colors represent the isosurfaces of the wavefunctions with the values of 0.002 and –0.002 atomic unit, respectively

Supplementary Note 1.

--MD Simulation details

-Supercell- for graphene/h-BN systems

Lengths of cell vectors in [angstrom]

La = 31.20, Lb = 31.20, Lc = 15.0

Angles between cell vec. in [deg.]

alpha = 90.0000, beta = 90.0000, gamma = 120.0000

-time step for MD simulations-

Using the Nose-Hoover thermostat technique at 300 K, the equation of motion was solved with a time step = 1.2 fs.

-Supercell for CNT-BNNT systems corresponds to Fig. 4h.

Lengths of cell vectors in [angstrom]

La = 40.00, Lb = 8.52, Lc = 40.00

Angles between cell vec. in [deg.]

alpha = 90.0000, beta = 90.0000, gamma = 90.0000

Supplementary References

1. Shikata, R., Suzuki, H., Hayashi, Y., Hasegawa, T., Shigeeda, Y., Inoue, H., Yajima, W., Kametaka, J., Maetani, M., Tanaka, Y., Nishikawa, T., Maeda, S., Hayashi, Y., Hada, M. Enhancement of the mechanical and thermal transport properties of carbon nanotube yarns by boundary structure modulation. *Nanotechnology* **33**, 235707 (2022).
2. Xiang, R., Inoue, T., Zheng, Y., Kumamoto, A., Qian, Y., Sato, Y., Liu, M., Tang, D., Gokhale, D., Guo, J., Hisama, K., Yotsumoto, S., Ogamoto, T., Arai, H., Kobayashi, Y., Zhang, H., Hou, B., Anisimov, A., Maruyama, M., Miyata, Y., Okada, S., Chiashi, S., Li, Y., Kong, J., Kauppinen, E. I., Ikuhara, Y., Suenaga, K., Maruyama, S. One-dimensional van der Waals heterostructures. *Science* **367**, 537–542 (2020).
3. Suzuki, H., Kishibuchi, M., Shimogami, K., Maetani, M., Nasu, K., Nakagawa, T., Tanaka, Y., Inoue, H., Hayashi, Y. *ACS Appl. Electron. Mater.* **3**, 3555–3566 (2021).
4. Tomio, Y., Suzuura, H., Ando, T. Interwall screening and excitons in double-wall carbon nanotubes. *Phys. Rev. B* **85**, 085411 (2012).
