# Supplementary material for: Photoinduced dynamics during electronic transfer from narrow to wide bandgap layers in one-dimensional heterostructured materials
Source: Nat Commun. 2024 May 30;15:4600. doi: 10.1038/s41467-024-48880-3 (PMC11139937; doi:10.1038/s41467-024-48880-3)
Supplement: Supplementary file 3 — Description of Additional Supplementary Files [file 41467_2024_48880_MOESM3_ESM.pdf]

Description of Additional Supplementary Files:

Supplementary Data 1: Initial_configuration_MD.xyz <= The initial configuration of MD for graphene/h-BN systems.

Supplementary Data 2: Final_configuration_MD.xyz <= The Final configuration of MD (4346.4 fs after the initial configuration) for graphene/h-BN systems.

Supplementary Data 3: Atomic_position_fig4a.xyz <= The atomic position used for Fig. 4a. The h-BN only configuration was picked from 2546.4 fs after the initial configuration of MD for graphene/h-BN systems.

Supplementary Data 4: Atomic_position_fig4b <= The atomic position used for Fig. 4b. The graphene only configuration was picked from 2546.4 fs after the initial configuration of MD for graphene/h-BN systems.

Supplementary Data 5: Atomic_position_fig4cde.xyz <= The atomic position used for Figs. 4c, d, and e. The configuration was picked from 2546.4 fs after the initial configuration of MD for graphene/h-BN systems. This configuration is also initial the configuration used for obtaining MSD for GS and ES in Fig. 4f.

Supplementary Data 6: Atomic_position_fig4h.xyz <= The atomic position used for Fig. 4h.
