# Supplementary material for: Photoinduced dynamics during electronic transfer from narrow to wide bandgap layers in one-dimensional heterostructured materials
Source: Nat Commun. 2024 May 30;15:4600. doi: 10.1038/s41467-024-48880-3 (PMC11139937; doi:10.1038/s41467-024-48880-3)
Supplement: Supplementary file 10 — Reporting Summary [file 41467_2024_48880_MOESM10_ESM.pdf]

nature portfolio

Corresponding author(s): Hiroo Suzuki, Satoshi Ohmura, Roman Bertoni, and Masaki Hada

Last updated by author(s): May 3, 2024

Reporting Summary

Nature Portfolio wishes to improve the reproducibility of the work that we publish. This form provides structure for consistency and transparency in reporting. For further information on Nature Portfolio policies, see our Editorial Policies and the Editorial Policy Checklist.

Statistics

For all statistical analyses, confirm that the following items are present in the figure legend, table legend, main text, or Methods section.

n/a	Confirmed	
☐	☒	The exact sample size (*n*) for each experimental group/condition, given as a discrete number and unit of measurement
☐	☒	A statement on whether measurements were taken from distinct samples or whether the same sample was measured repeatedly
☒	☐	The statistical test(s) used AND whether they are one- or two-sided<br>*Only common tests should be described solely by name; describe more complex techniques in the Methods section.*
☒	☐	A description of all covariates tested
☒	☐	A description of any assumptions or corrections, such as tests of normality and adjustment for multiple comparisons
☐	☒	A full description of the statistical parameters including central tendency (e.g. means) or other basic estimates (e.g. regression coefficient) AND variation (e.g. standard deviation) or associated estimates of uncertainty (e.g. confidence intervals)
☒	☐	For null hypothesis testing, the test statistic (e.g. *F*, *t*, *r*) with confidence intervals, effect sizes, degrees of freedom and *P* value noted<br>*Give P values as exact values whenever suitable.*
☒	☐	For Bayesian analysis, information on the choice of priors and Markov chain Monte Carlo settings
☒	☐	For hierarchical and complex designs, identification of the appropriate level for tests and full reporting of outcomes
☒	☐	Estimates of effect sizes (e.g. Cohen's *d*, Pearson's *r*), indicating how they were calculated

Our web collection on statistics for biologists contains articles on many of the points above.

Software and code

Policy information about availability of computer code

Data collection	For this study, following software was used for data collection: Labview.
Data analysis	For this study, following software was used for data analysis and plotting: Excel, Origin, Matlab.

For manuscripts utilizing custom algorithms or software that are central to the research but not yet described in published literature, software must be made available to editors and reviewers. We strongly encourage code deposition in a community repository (e.g. GitHub). See the Nature Portfolio guidelines for submitting code & software for further information.

Data

Policy information about availability of data

All manuscripts must include a data availability statement. This statement should provide the following information, where applicable:

- Accession codes, unique identifiers, or web links for publicly available datasets
- A description of any restrictions on data availability
- For clinical datasets or third party data, please ensure that the statement adheres to our policy

Source data of this manuscripts are provided in Figshare with the identifier "https://doi.org/10.6084/m9.figshare.25728837". All relevant experimental and computational data within the manuscript are available from the corresponding author upon request. The computational codes within the manuscript are available from the corresponding author upon request. The first principles calculations in this study were performed based on the TDDFT method. The source code for the

calculations is available as QXMD code available in https://usccacs.github.io/QXMD/index.html. (Permanent link to the version of code/repository for the TDDFT method can also found in https://github.com/ElsevierSoftwareX/SOFTX_2019_49).

Research involving human participants, their data, or biological material

Policy information about studies with human participants or human data. See also policy information about sex, gender (identity/presentation), and sexual orientation and race, ethnicity and racism.

Reporting on sex and gender	N/A
Reporting on race, ethnicity, or other socially relevant groupings	N/A
Population characteristics	N/A
Recruitment	N/A
Ethics oversight	N/A

Note that full information on the approval of the study protocol must also be provided in the manuscript.

Field-specific reporting

Please select the one below that is the best fit for your research. If you are not sure, read the appropriate sections before making your selection.

☐ Life sciences ☐ Behavioural & social sciences ☒ Ecological, evolutionary & environmental sciences

For a reference copy of the document with all sections, see nature.com/documents/nr-reporting-summary-flat.pdf

Ecological, evolutionary & environmental sciences study design

All studies must disclose on these points even when the disclosure is negative.

Study description	Our research is based on studying physical property of materials. More specifically, ultrafast time-resolved measurements were performed on one-dimensional heterostructure of carbon nanotubes and boron nitride nanotubes. We repeated the measurements few time to ensure the reproducibility of the time-resolved measurements.
Research sample	The samples are few-walled carbon nanotubes, few-walled carbon nanotubes covered with few-walled boron nitride nanotubes and few-walled carbon nanotubes covered with multi-walled boron nitride nanotubes. All samples were synthesized by H. Suzuki.
Sampling strategy	All data are reported in the manuscript.
Data collection	For the data collection, we used automated experimental setups. For examples, time-resolved electron diffraction measurements were based on Labview software. The other experiments were based on commercial programs. H. Suzuki is in charge of TEM measurements, Raman spectroscopy. M. Hada is in charge of time-resolved electron diffraction measurements and absorption spectrometry. R. Bertoni is in charge of transient optical absorption experiments.
Timing and spatial scale	Time-resolved electron diffraction measurements were performed from September 2020 to December 2022. Time-resolved electron diffraction experiments for revision were performed from December 2023 to January 2024. Transient optical absorption experiments were performed from February 2023 to October 2023.
Data exclusions	No data was excluded.
Reproducibility	We confirmed reproducibility for the time-resolved measurements.
Randomization	N/A
Blinding	N/A

Did the study involve field work? ☐ Yes ☒ No

Reporting for specific materials, systems and methods

We require information from authors about some types of materials, experimental systems and methods used in many studies. Here, indicate whether each material, system or method listed is relevant to your study. If you are not sure if a list item applies to your research, read the appropriate section before selecting a response.

Materials & experimental systems

n/a	Involved in the study
☒	☐ Antibodies
☒	☐ Eukaryotic cell lines
☒	☐ Palaeontology and archaeology
☒	☐ Animals and other organisms
☒	☐ Clinical data
☒	☐ Dual use research of concern
☒	☐ Plants

Methods

n/a	Involved in the study
☒	☐ ChIP-seq
☒	☐ Flow cytometry
☒	☐ MRI-based neuroimaging

Plants

Seed stocks	N/A
Novel plant genotypes	N/A
Authentication	N/A
